# Cerebrospinal fluid CD4+ T cell infection in humans and macaques during acute HIV-1 and SHIV infection

**DOI:** 10.1371/journal.ppat.1010105

**Published:** 2021-12-07

**Authors:** Vishakha Sharma, Matthew Creegan, Andrey Tokarev, Denise Hsu, Bonnie M. Slike, Carlo Sacdalan, Phillip Chan, Serena Spudich, Jintanat Ananworanich, Michael A. Eller, Shelly J. Krebs, Sandhya Vasan, Diane L. Bolton, the RV254/SEARCH010 and RV304/SEARCH013 Study Teams

**Affiliations:** 1 Military HIV Research Program, Walter Reed Army Institute of Research, Silver Spring, Maryland, United States of America; 2 Henry M. Jackson Foundation for the Advancement of Military Medicine, Bethesda, Maryland, United States of America; 3 Department of Retrovirology, Armed Forces Research Institute of Medical Sciences, Bangkok, Thailand; 4 Institute of HIV Research and Innovation, Bangkok, Thailand; 5 Department of Neurology, Yale University, New Haven, Connecticut, United States of America; Emory University, UNITED STATES

## Abstract

HIV-1 replication within the central nervous system (CNS) impairs neurocognitive function and has the potential to establish persistent, compartmentalized viral reservoirs. The origins of HIV-1 detected in the CNS compartment are unknown, including whether cells within the cerebrospinal fluid (CSF) produce virus. We measured viral RNA+ cells in CSF from acutely infected macaques longitudinally and people living with early stages of acute HIV-1. Active viral transcription (spliced viral RNA) was present in CSF CD4+ T cells as early as four weeks post-SHIV infection, and among all acute HIV-1 specimens (N = 6; Fiebig III/IV). Replication-inactive CD4+ T cell infection, indicated by unspliced viral RNA in the absence of spliced viral RNA, was even more prevalent, present in CSF of >50% macaques and human CSF at ~10-fold higher frequency than productive infection. Infection levels were similar between CSF and peripheral blood (and lymph nodes in macaques), indicating comparable T cell infection across these compartments. In addition, surface markers of activation were increased on CSF T cells and monocytes and correlated with CSF soluble markers of inflammation. These studies provide direct evidence of HIV-1 replication in CD4+ T cells and broad immune activation in peripheral blood and the CNS during acute infection, likely contributing to early neuroinflammation and reservoir seeding. Thus, early initiation of antiretroviral therapy may not be able to prevent establishment of CNS viral reservoirs and sources of long-term inflammation, important targets for HIV-1 cure and therapeutic strategies.

## Author summary

Neurological pathologies are associated with HIV-1 infection and remain common in the ongoing AIDS epidemic. Despite the advent of successful viremia suppression by anti-retroviral therapy, increased life expectancies and co-morbidities have led to higher prevalence of milder forms of neurocognitive dysfunction. How HIV-1 causes neurocognitive dysfunction is currently unclear, though it is widely believed that viral replication within the central nervous system (CNS) prior to therapy triggers these detrimental processes. The appearance of HIV-1 in the cerebrospinal fluid during the earliest stages of infection suggests that these processes may begin very early. Here, we use novel techniques to probe cells for viral infection during the first few weeks of infection in the CNS of humans and animals to determine the source of this virus. We found HIV-1 replication in T cells in the cerebrospinal fluid during this early window. In addition, infected T cells were present at similar frequencies in the CNS and other anatomic compartments, suggesting equilibration of T cell infection levels across these sites and potential for establishment of long-term reservoirs in the CNS. Our study provides new insights to the early events of viral entry and replication in the CNS with implications for subsequent viral persistence and neuronal injury.

## Introduction

Successful viremia suppression by anti-retroviral therapy (ART) has significantly reduced neurological pathologies associated with HIV-1 infection. However, increased life expectancies and co-morbidities in the ART era has led to higher prevalence of milder forms of HIV-related neurocognitive dysfunction [1,2], which remain a challenge for people living with HIV-1 (PLWH). The mechanisms underlying the neurocognitive dysfunction associated with HIV-1 are complex and currently unclear [3,4], though it is widely believed that HIV-1 replication within the central nervous system (CNS) prior to therapy initiation triggers neuropathogenesis. Overall neurological dysfunction in treated infection is thought to mainly result from widespread inflammation in the CNS in response to the virus rather than direct viral infection and replication [5], though a contribution of viral infection/persistence to neurologic injury during ART is an area of intense study. The very early appearance of HIV-1 RNA in the CSF of acutely infected PLWH suggests that these processes may begin within the first weeks of infection [6,7]. The cellular origin of this virus and the extent to which it represents independent CNS viral replication is unknown. Importantly, early viral replication in the CNS may also establish viral reservoirs that persist during ART and pose a barrier to HIV-1 cure.

There remains debate in the field as to how HIV-1 enters the CNS compartment and the relative role of myeloid versus CD4+ T cells in this process. Infected microglia and macrophages in the brain along with aberrant inflammatory responses among these resident cell populations are established drivers of neuropathogenesis in chronic infection [8–12]. However, evidence of CSF inflammation and viral detection within two weeks of HIV-1 infection along with very early compartmentalization of HIV-1 sequences in the CNS indicate an early phase of viral entry when CD4+ T cells are more likely to be involved [7,13–15]. This is supported by CSF transmitted viral variants early in human HIV-1 infection that are T cell-tropic and inflammatory infiltrates in the brain parenchyma of acute CCR5-tropic SHIV-infected macaques comprised predominantly of T cells [15,16]. Gradual emergence of viral lineages with greater macrophage tropism in human CSF months post-infection suggests that selection for non-T cell entry occurs following CNS invasion [15]. Recent studies characterizing CSF virus during ART-suppressed chronic infection also implicate T cells as the origin of HIV-1 RNA detected in the CNS at this later stage [17,18]. Direct evidence of CNS cells harboring virus and supporting viral replication is necessary to delineate early events in CNS seeding.

To define the early cellular targets of HIV-1 replication in the CNS and the association with CNS cellular inflammation and activation, we assessed CSF cell populations for viral RNA and phenotypic changes during acute infection using a novel combined flow cytometric and PCR approach developed for samples with low cellular input. CSF samples from humans and SHIV-infected rhesus macaques were interrogated to study these events in two complementary models, at peak viremia in humans and longitudinal time points in macaques. The SHIV strain was selected for its prior characterization in macaque CNS during acute infection and its non-neurovirulent phenotype, distinct from accelerated neurovirulent SIV models that result in severe CNS disease more rapidly than seen in humans [19–21]. Assays specific for spliced and genomic viral RNA were used to distinguish different stages of cellular infection, including cells actively replicating virus. We compared the infected cell burden in CSF to that in peripheral blood (PB) and lymph nodes to discern compartmentalized replication across anatomic sites. Lastly, we measured the immunologic activation state of CSF cell populations and soluble indices during acute infection. Our findings clarify early host cell infection events and cellular inflammatory responses occurring in the CNS during acute HIV-1 infection.

## Methods

### Ethics statement

All animal *in vivo* procedures were carried out in accordance with institutional, local, state, and national guidelines and laws governing research in animals including the Animal Welfare Act. Animal protocols and procedures were reviewed and approved by the Animal Care and Use Committee of both the US Army Medical Research and Development Command (USAMRDC, Maryland, USA) Animal Care and Use Review Office as well as the Institutional Animal Care and Use Committee (IACUC) of Bioqual, Inc. (Maryland, USA, protocol number 14-B077) or the IACUC of the Armed Forces Research Institute of Medical Science (AFRIMS; Bangkok, Thailand, protocol number PN 13–07) where the non-human primates were housed. Bioqual, Inc., AFRIMS, and the USAMRDC are accredited by the Association for Assessment and Accreditation of Laboratory Animal Care and are in full compliance with the Animal Welfare Act and Public Health Service Policy on Humane Care and Use of Laboratory Animals. The clinical study protocols were approved by the institutional review boards of Chulalongkorn University (Bangkok, Thailand) and the Walter Reed Army Institute of Research (Silver Spring, MD). All participants provided written informed consent to participate in the study.

### Animals and SHIV infection

Eighteen colony-bred adult Indian-origin male and female rhesus macaques were infected with dual-tropic SHIV-1157ipd3N4 administered intrarectally or intravaginally (Bioqual animal protocol 14-B077, N = 12; AFRIMS protocol PN13-07, N = 6) [16,22]. Challenge dose ranged from AID50 to AID80 (14-B077) and 3.9–78 x 10^6^ genomic RNA copies (PN13-07), administered in 1 mL. Peripheral blood mononuclear cells (PBMC), lymph node mononuclear cells (LNMC), and CSF cell pellet specimens were collected longitudinally following infection and viably cryopreserved. Post-infection sampling occurred at weeks 4, 8, and 12 (14-B077) or at week 12 (PN13-07), with additional PBMC collection 2–3 weeks PI in 14-B077. CSF cells originated from 6–12 mL of CSF yielding 760–680,000 mononuclear cells. Six uninfected animals served as negative controls.

### Human specimens

Eleven male participants from an acute HIV-1 infection cohort in Bangkok, Thailand, RV254 / SEARCH010 (NCT00796146, Table 1) [23] were selected for inclusion in this study based on presentation in Fiebig stages III (N = 9) and IV (N = 2) and available CSF and PBMC specimens [24]. Cell specimens were collected and cryopreserved at study enrollment when all individuals were ART naive [25]. CSF cells originated from 8–9 mL of CSF and ranged in yield from 3,000–350,000 cells. Samples from five age- and gender-matched Thai people living without HIV-1 (PWOH) were also included (RV304 / SEARCH013, NCT01397669). The investigators adhered to the policies for protection of human subjects as prescribed in Army Regulation 70–25.

**Table 1:** Demographic and clinical characteristics for people living with acute HIV Infection and for people without HIV.

Study	Participant ID	Fiebig Stage	Subtype	Plasma HIV RNA (copies/mL, log10)	CSF HIV RNA (copies/mL, log10)	Age	Gender
RV254	9104	III	CRF01_AE	7.68	5.19	23	M
RV254	5940	III	CRF01_AE	7.33	3.99	25	M
RV254	6803	III	CRF01_AE	7.27	5.43	21	M
RV254	7878	III	CRF01_AE	6.96	3.20	41	M
RV254	7268*	III	CRF01_AE	6.95	5.22	23	M
RV254	5365*	III	CRF01_AE	6.78	3.11	30	M
RV254	6130*	III	CRF01_AE	6.66	5.08	27	M
RV254	4324*	III	CRF01_AE	6.50	4.15	22	M
RV254	6799*	III	CRF01_AE	6.47	4.10	22	M
RV254	4153	IV	CRF01_AE	6.70	4.33	29	M
RV254	7188*	IV	CRF01_AE	6.20	3.60	24	M
RV304	1874*	N/A	N/A	N/A	N/A	31	M
RV304	1592*	N/A	N/A	N/A	N/A	30	M
RV304	1713*	N/A	N/A	N/A	N/A	33	M
RV304	1720	N/A	N/A	N/A	N/A	20	M
RV304	1845	N/A	N/A	N/A	N/A	31	M

*Participants assessed for cell-associated HIV-1 RNA.

N/A, not applicable

### Flow cytometry and cell sorting

Cryopreserved PBMC and CSF cell samples were thawed and stained with fluorescent conjugated antibodies specific for lineage and activation markers (BD Biosciences unless otherwise noted). Rhesus macaque specimens were stained with the following: CD14-APC (M5E2), CD16-PE-Cy7 (3G8), CD3-AL700 (SP34-2), CD20-PE-CF594 (2H7), CD4-BV605 (OKT4, Biolegend), CD8a-BV785 (RPA-T8), CD95-BV421 (DX2), and CD28-PE-Cy5 (CD28.2); and markers of activation: CD38-PE (OKT10), CD69-BUV395 (FN50), CD169-FITC (7–239, AbD Serotec), CD86-BV650 (IT2.2), and HLA-DR-PERCP CY5.5 (L243). Monocyte activation was measured using CD169 rather than CD16/CD14 co-expression due to greater resolution of CD169 staining on CSF monocytes. Briefly, cells were stained with LIVE/DEAD Fixable Aqua Dead Cell Stain Kit (Thermo Fisher Scientific) to exclude dead cells, washed, and surface stained with the antibody cocktail. Human specimens were stained with the following: CD14-PB (M5E2), CD16-PE Cy7 (3G8), CD3-BV711 (UCHT1, Biolegend), CD4-BUV496 (SK3), CD8-BV785 (RPA-T8, Biolegend), CD45RO-BV650 (UCHL1, Biolegend), CCR7-FITC (G043H7, Biolegend), CD20-BV510 (2H7, Biolegend); and markers of activation: CD127-BV605 (HIL-7R-M21), LAG3-APC (R&D Systems), TIM3-APC (F38-2E2, Biolegend), PD1-A647 (EH12.1), TIGIT-APC (MBSA43, Thermo Fisher Scientific), CD69-BUV395 (FN50), CD38 BUV737 (HB7), HLA DR-APC H7 (L243), CD29-AL700 (TS2/16, Biolegend), CD49d-PE Cy5 (9F10, Biolegend), CD169-PE (7–239), and CD163-PerCPCy5.5 (GH1/61, Biolegend). Viable monocytes (CD14+, CD16+, or CD14+CD16+), CD4+ T cells, CD8+ T cells or cells negative for lineage markers (CD14-CD3-CD4-CD8-) were sorted on a 5-laser FACS Aria II SORP (BD Biosciences). PBMC and LNMC were sorted into a 96-well PCR plate (BioExpress) in a serial 3-fold limiting dilution format ranging from 3–1000 cells in replicate or for single-cell analysis as previously described [26]. CSF cells were sorted at a single dilution ranging from 1–1000 cells in replicate into 200 μl PCR tubes (Eppendorf); dilutions were customized for each specimen to ensure multiple replicates for each population. PCR plates and tubes were maintained on pre-chilled aluminum plates and ice respectively. Cells were sorted directly into qRT-PCR buffer for immediate lysis.

### cDNA synthesis and quantitative gene expression

RNA from FACS-sorted cells lysed in 10 μl of Superscript III Platinum One-Step qRT-PCR buffer (Cells Direct 1X reaction mix, Superscript III RT Platinum Taq and 0.2x gene-specific TaqMan assay mix) was reverse transcribed and pre-amplified on a GeneAmp PCR System 9700 (ThermoFisher Scientific) using the following conditions: 50°C for 15 min, 95°C for 2 min and 18 rounds of 95°C for 15 seconds and 60°C for 4 min [27]. Quantitative PCR (qPCR) for viral genes was performed on the QuantStudio 6 Flex Real-Time PCR System (Thermo Fisher Scientific). SHIV-1157ipd3N4 mRNA-specific primers and probes were optimized on bulk cell RNA extracts from PHA-stimulated rhesus primary cells infected *in vitro* with SHIV-1157ipd3N4. Qualified assays exhibited linear amplification on serially diluted cDNA template [27], with sensitivity at cell equivalent concentrations as low as three cells. Unspliced viral genomic RNA was measured using the previously described SIV *gag* assay: forward: 5’- GTC TGC GTC ATY TGG TGC ATT C-3’; reverse: 5’-CAC TAG RTG TCT CTGC ACT ATY TGT TTT G-3’ and probe: 5’- CTT CYT CAG TRT GTT TCA CTT TCT CTT CTG CG-3’ [26,28]. Spliced SHIV *env* mRNA was measured using the following: forward: 5’-CGG CGT GAG GAG CGG-3’; reverse: 5’-AGT CTG ACT GTT CTG ATG AGC-3’; probe: 5’-CCT CCG GTT GCA GGA AGA AGC GGA GA-3’ (Integrated DNA Technologies). Unspliced CRF01_AE HIV-1 RNA was measured using assays previously described [29]. as follows: 1) *gag*: forward: 5’- CAT TAG AAG ARA TGA TGA CAG CAT-3’, reverse: 5’-GCT CAT TGC CTC RGC YAA AAC-3’, probe: 5’-AGT RGG AGG ACC TRG CCA TAA RGC AAG-3’; 2). spliced CRF01_AE HIV-1 *env*: forward: 5’-CTG MGG TGC ACA CAG CAA GA-3’, reverse: 5’- GAG GAG KTC YTC GTC GGT G-3’, probe: 5’- TTC CGC TTC TTC CAG TCG CCG CTC T-3’; and 3) total HIV-1 RNA *LTR* (adapted from [30]): forward: 5’-CTG GGT CTC TCT DGT TAG AC-3’, reverse: 5’-CTG AGG GAT CTC TAG TTA CC-3’, probe: 5’CAC TCA AGG CAA GCT TTA TTG AGG C-3’. Probe modifications were as follows: 5’ 6-FAM, 3’ Iowa Black FQ, Int ZEN). *CXCL10* expression was measured using a TaqMan assay (Rh02788358_m1), ThermoFisher Scientific) using the 2^–ΔΔCt^ method relative to *GAPDH* (Rh02621745_g1). Cell surface-bound virion-derived *gag* vRNA was measured by incubation of PBMC in 0.05% trypsin for 5 min at room temperature to cleave CD4 from the cell surface, washed in 15% fetal bovine serum once and PBS twice, followed by surface staining and limiting dilution FACS sorting of memory CD4 T cells (CD8-negative) and CRF01_AE HIV-1 *gag* RT-qPCR.

### Soluble protein biomarker concentration

Levels of soluble biomarkers were measured in CSF and plasma via a combination of Luminex-based custom assays and ELISAs, as per manufacturers’ protocol and as described previously [31]. IP-10/CXCL10 and IL-10 were measured using the Luminex based Milliplex Map Human Cytokine / Chemokine Panel (Millipore Sigma, St. Louis MO). Soluble CD163 was measured via the Bio-Plex Pro Human Inflammation Panel 1 (Bio-Rad Laboratories, Hercules CA). Neopterin was measured by chemiluminescent detection ELISA (Genway Biotech, San Diego CA). Data was collected on a FlexMap 3D reader (Bio-Rad Laboratories, Hercules CA) or VersaMax plate reader (Molecular Devices, Sunnyvale CA) and analyzed in Prism v.9 for Mac OS X (GraphPad, La Jolla CA) using 4-parameter fit standard curves.

### Statistics

Poisson distribution statistics were used to estimate the frequency of infected cells harboring *gag* and/or *env* mRNA in replicate limiting diluted PBMC and LNMC as described previously [26]. For CSF, the infected cell frequency was estimated by conservative assignment of one positive cell to each replicate in which viral mRNA was detected. Wilcoxon matched-pairs rank test was used to compare infected cell frequency between tissues. Flow cytometry data were analyzed in FlowJo v9.9.6. Mann-Whitney test (*P<*0.05) was used to assess differential surface protein expression. Spearman correlation was used to measure associations between parameters.

## Results

### Viral and CSF cellular dynamics in acute SHIV infection

**Fig 1:**
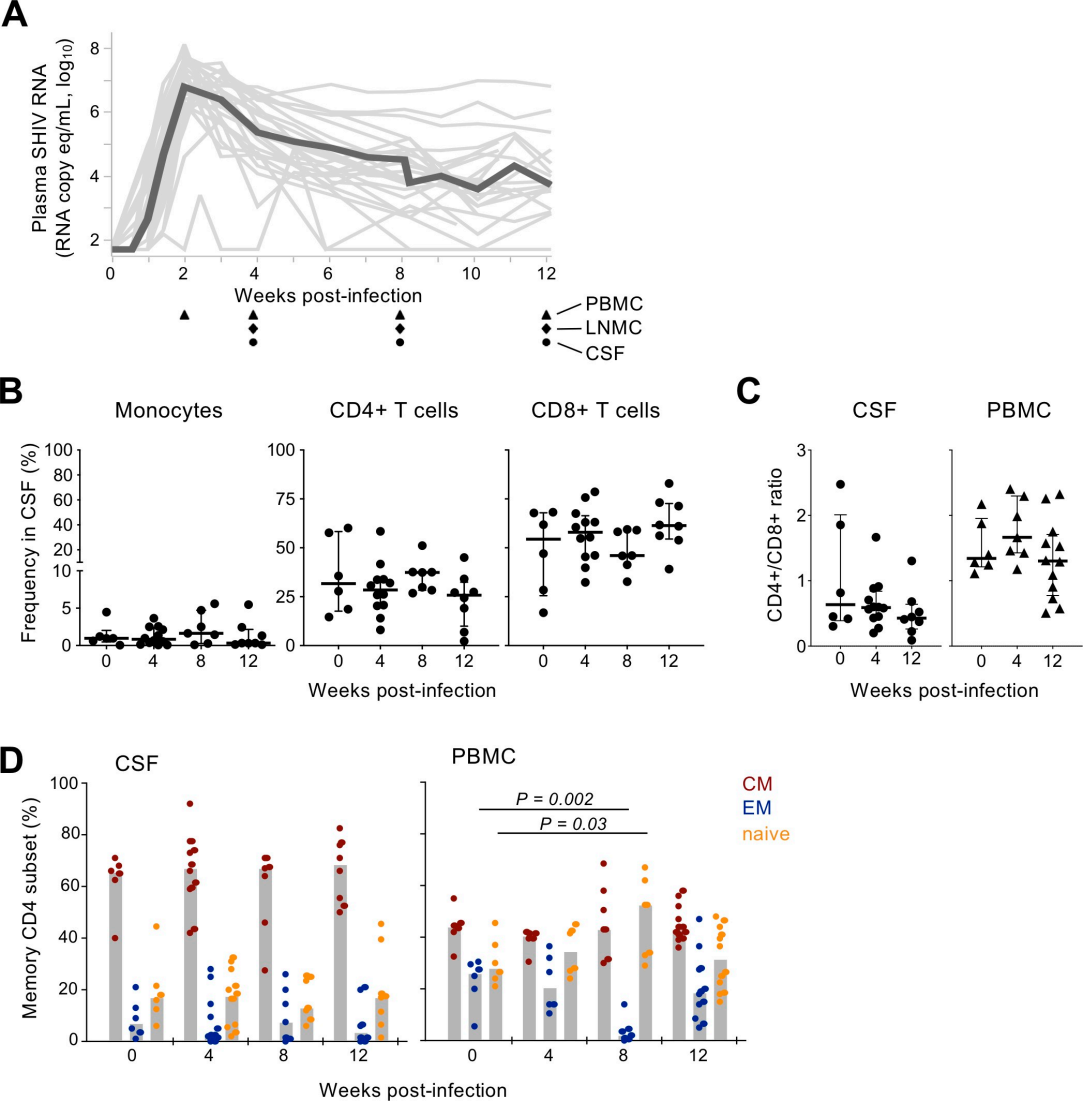
Plasma viremia and CSF cellular composition in acutely SHIV-infected macaques. (A) Longitudinal plasma viral RNA following SHIV-1157ipd3N4 infection in rhesus macaques (N = 18). Data for individual animals is shown in gray and the black trend line depicts the median value. Biospecimen collections are indicated by symbols at bottom. (B) The frequency of T cell subsets and monocytes in CSF determined by flow cytometry is plotted over time PI. T cell subset frequency was calculated as the percentage of all T cells; monocytes as the percentage of all viable CSF cells. (C) The CD4+/CD8+ T cell ratio in CSF (left) and PBMC (right) is shown in uninfected and SHIV-infected macaques 4 and 12 weeks PI. (D) The proportion of memory CD4+ T cell subsets in CSF (left) and PBMC (right) was assessed by CD28 and CD95 staining and flow cytometry. The frequency of central memory (CM; CD95+ and CD28+), effector memory (EM; CD95+ and CD28-negative), and naive (CD95-negative) is shown over time.

To study the dynamics of cellular inflammatory processes and cell-associated viral replication within the nervous system during acute infection, 18 rhesus macaques were infected intrarectally or intravaginally with the well-characterized SHIV-1157ipd3N4, which encodes an R5-tropic subtype C HIV-1 *env* also able to infect monocyte-derived macrophages [16,22]. Infection with this virus recapitulates many aspects of acute HIV-1 infection, including early CSF viral detection and inflammation [16]. Peak plasma viremia occurred two weeks post-infection (PI) and viremia was sustained in most animals for three months (**Fig 1A**). CSF SHIV RNA was detected in cell-free CSF supernatant in 4 of 12 animals assessed 12 weeks PI and ranged from 6–722 copies/mL as previously reported [16].

Given dynamic cellular processes that occur during acute infection, including CSF pleocytosis in HIV-1 infection [15,32], we characterized CSF mononuclear cell composition throughout early SHIV infection. Cryopreserved CSF cells collected 4, 8, and 12 weeks PI from a subset of the animals (N = 7–12) and six uninfected controls were thawed and stained with lymphocyte and myeloid cell lineage markers and analyzed by flow cytometry (**S1A Fig**). PBMC were analyzed in a similar manner for comparison **(S1B Fig).** The relative abundance of monocytes in the CSF was similar between uninfected animals and following SHIV infection, as was the frequency of CD4+ and CD8+ T cells (**Fig 1B**). The CD4/CD8+ T cell ratio in both CSF and PB also did not change significantly (**Fig 1C**). CSF CD4+ T cells were predominantly of a central memory phenotype (median ~65%) with limited effector memory cells (median <10%), and the memory subset composition was unaltered throughout acute infection (**Fig 1D**). In contrast, CD4+ T cells circulating in PB comprised a more balanced ratio of memory and naïve subsets and suffered transient effector memory cell loss 8 weeks PI, consistent with depletion of this population by CCR5-tropic SIV and HIV-1 [33,34]. These data indicate that the CSF cellular makeup is distinct from PB and may undergo modest changes during acute SHIV infection.

### SHIV replication in CSF CD4+ T cells of acutely infected macaques

**Fig 2:**
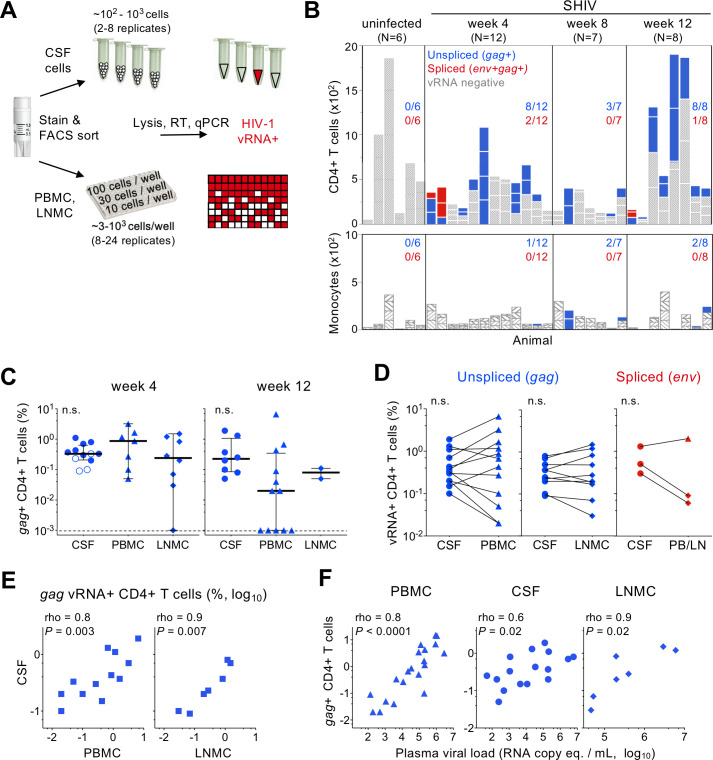
Cell-associated SHIV RNA during acute infection. (A) Schematic of workflow used to isolate mononuclear cell populations and estimate infected cell frequency in CSF (top) and PBMC or LNMC (bottom) directly ex vivo from SHIV-infected macaques. (B) Viral RNA RT-qPCR positivity among FACS sorted CD4+ T cells (top) and monocytes (bottom) in CSF of uninfected and SHIV-infected macaques 4, 8, and 12 weeks PI. The number of cells analyzed for each animals is indicated on the y-axis, with stacked bars reflecting the size of each sort replicate. Bar coloring indicates positivity for unspliced only (blue) or spliced (red) SHIV RNA; viral RNA-negative replicates are colored gray. Fraction of animals with SHIV RNA+ cells is indicated for each time point. (C) SHIV *gag* RNA+ CD4+ T cell frequency is shown for CSF, PBMC, and LNMC 4 and 8 weeks PI. Open symbols depict the limit of detection in samples where no vRNA was detected. Significant differences between tissues was assessed using Mann-Whitney test. (D) Paired analysis comparing the infected CD4+ T cell frequency between matched CSF and either PBMC or LNMC is shown. (E) Correlation between *gag* vRNA+ infected CD4+ T cell frequency between CSF and PBMC (left) or LNMC (right) is shown. (F) Correction between plasma viremia and *gag* vRNA+ infected CD4+ T cell frequency (percent, log10) in PBMC (left), CSF (middle), and LNMC (right) is shown. Rho and p-value indicate Spearman correlation.

Though detection of SHIV RNA in the cell-free component of CSF was limited in this SHIV model, we hypothesized that cells within the CNS harbored virus based on prior observations of viral RNA+ cells in the brain parenchyma of SHIV-infected macaques [16]. To probe for cell-associated virus in the CSF and identify cell targets, CD4+ T cells, monocytes, and CD8+ T cells were FACS sorted in replicate and analyzed by viral RNA (vRNA) RT-qPCR directly *ex vivo* (**Fig 2A**). Cellular vRNA was selected for analysis in an effort to capture active viral transcription, which is not reflected by integrated DNA or intact proviral DNA measurements. SIV *gag* was used as a marker of unspliced vRNA, which can be derived from virions due to its presence in the viral genome [26,28]. To detect active replication, we developed and validated an *env/vpu/nef* mRNA assay (“*env”*) specific for these spliced transcripts of SHIV-1157ipd3N4 (**S2 Fig)**. Previous analysis of SIV-infected CD4+ T cells using *gag* and a related SIV-specific *env* assay demonstrated a ~100-fold increase in genomic vRNA in *env*+ cells relative to *env*-negative *gag*+ cells, consistent with viral replication in cells identified by the spliced *env* assay [26]. vRNA was present in the majority of the CSF CD4+ T cell specimens, with 75% of animals positive at week 4 PI (N = 12) (**Fig 2B**). *gag* vRNA in the absence of *env* predominated, indicating widespread non-productive infection (e.g. early, latent, abortive). Transcriptionally active or productive CD4+ T cell infection (*env+gag+)* was more limited and detected in CSF of two of twelve animals, those with the highest plasma viremia 4 weeks PI. The median percentage of infected CD4+ T cells was 0.4% for both *gag*+ and *env+gag+* cells among animals with values above the limit of detection. Analysis of week 12 CSF, which was prioritized only for animals with detectable week 12 PBMC CD4+ T cell infection (described below), demonstrated *gag* vRNA+ infection in all animals (N = 8; median 0.2% *gag+*) and productive infection in one animal (1.3% *env*+). Productive infection was not observed in CSF monocytes, while *gag* was present infrequently (**Fig 2B**, bottom), though limited monocyte yields (~20% that of CD4+ T cells) constrained sampling depth. No vRNA was detected in any samples from uninfected controls and vRNA was exceedingly rare among CD8+ T cells from SHIV-infected animals (**S3A and S3B Fig**).

Infected CD4+ T cell frequency was estimated for each animal with vRNA+ CD4+ T cells detected in CSF. *gag* vRNA*+* infected cells ranged from 0.2–1.1% at week 4 PI and 0.05% - 1.9% at week 12 (**Fig 2C**). To compare the CSF infected cell burden to other anatomical sites, memory CD4+ T cells and monocytes from contemporaneous PBMC and lymph node mononuclear cell (LNMC) specimens were analyzed in parallel (**Fig 2A**). PBMC and LNMC CD4+ T cell infection levels were similar to that of CSF at week 4 PI (0.05%-1.6% and 0.03%-1.5%, respectively) and week 12 PI (0.02%-6.6% and 0.1%-0.2%, respectively). Comparing matched specimens within an animal, neither non-productively nor productively infected CD4+ T cell frequencies differed between CSF, PBMC and LNMC (**Fig 2D**), indicating similar infection levels across these compartments. Moreover, *gag* vRNA+ infected cell frequencies were positively correlated between CSF and PBMC (rho = 0.8, *P =* 0.003), and CSF and LNMC (rho = 0.9, *P =* 0.007) (**Fig 2E**). Infected CD4+ T cells in PBMC, CSF and LNMC all also positively correlated with plasma viral load (rho = 0.8, *P<*0.001; rho = 0.6, *P =* 0.02; rho = 0.9, *P =* 0.02, respectively) (**Fig 2F**). Thus *gag+* SHIV-infected CD4+ T cells are as prevalent in the CSF as they are in circulation and lymph nodes and their burden across these anatomical sites tracks closely with systemic viremia.

### Lymphocyte activation in CSF and PB during acute SHIV infection

**Fig 3:**
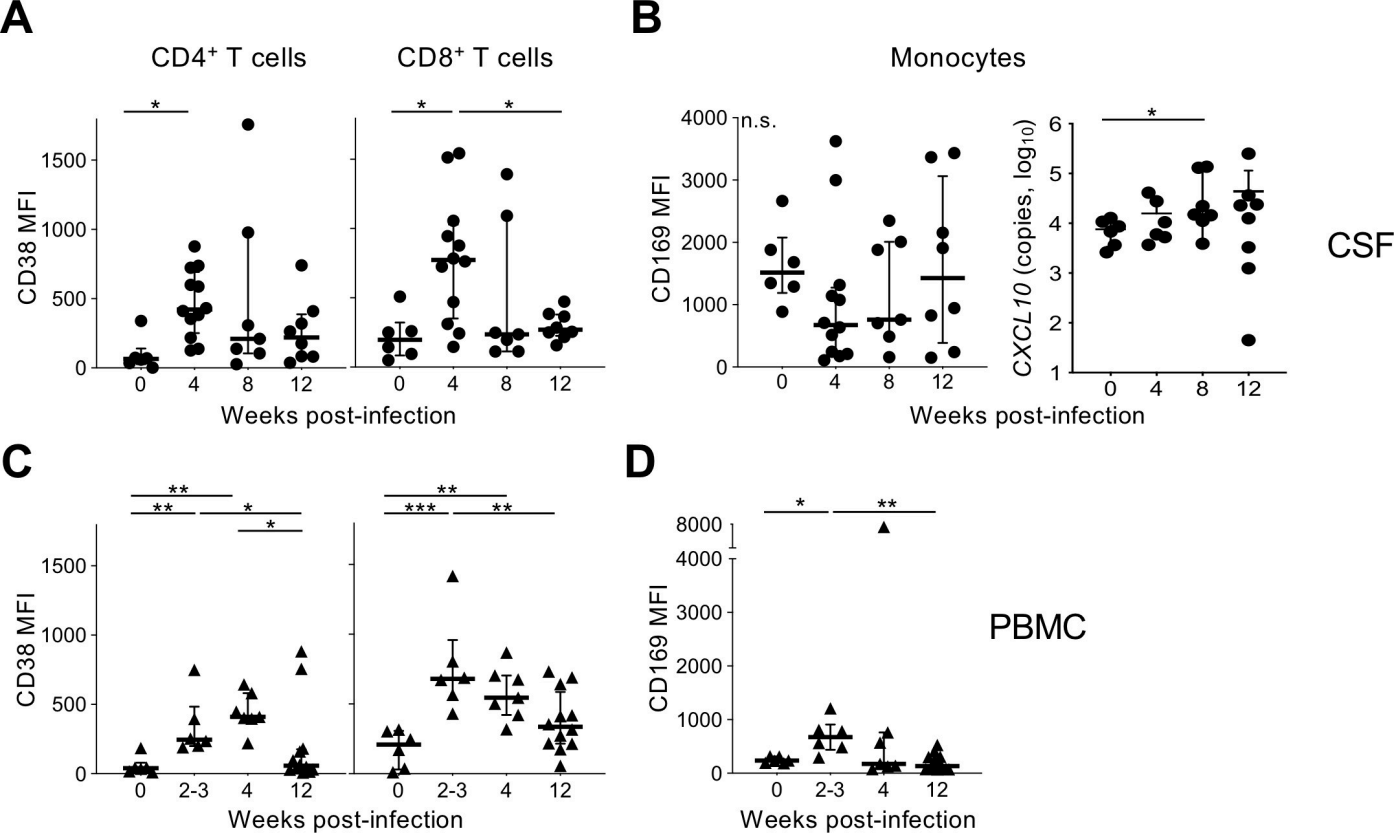
Cellular activation in CSF and PBMC during acute SHIV infection. T cell and monocyte activation assessed by surface staining flow cytometry for CD38 and CD169, respectively, following SHIV infection in macaques. (A) CSF CD4+ and CD8+ T cell CD38 expression, reported as the median fluorescent intensity (MFI), is shown at the indicated weeks PI. (B) CSF monocyte CD169 MFI and *CXCL10* gene expression (copies per 10^6^ cells) measured by RT-qPCR is shown at the indicated weeks PI. (C) PBMC CD4+ and CD8+ T cell CD38 surface protein expression and (D) PBMC monocyte CD169 expression is shown at the indicated weeks PI. Bars and whiskers indicate median and interquartile range values, respectively. Significant differences by Mann-Whitney test are indicated as follows: *, P <0.05; **, P <0.01; ***, P <0.0001.

Since cellular activation can mediate CNS inflammation and may increase susceptibility to viral replication[35], we examined the activation status of CSF cells during acute SHIV infection by surface marker staining and flow cytometry. CD38 expression increased on both CD4+ and CD8+ T cells in CSF 4 weeks PI (*P<*0.05) (**Fig 3A**). T cell activation was transient and largely resolved by week 12. CSF monocytes did not show evidence of activation based on surface expression of the cell adhesion molecule and activation marker CD169 (**Fig 3B**), though expression of *CXCL10*, which encodes the chemoattractant IP-10, was elevated at week 8. In a parallel analysis of PBMC, similar results were observed for CD4+ and CD8+ T cells, with activation apparent as early as 2–3 weeks PI, sustained at week 4, and diminished by week 12 (**Fig 3C**). Blood monocyte activation was also observed 2–3 weeks PI, but mostly resolved by weeks 4 and 12, consistent with lack of CSF monocyte activation at these times (**Fig 3D**). T cell infection levels did not correlate with cellular activation. These data indicate pronounced activation of T cells in both CSF and PB during the first several weeks of acute SHIV infection, while monocyte activation was more transient and limited to blood.

### HIV-1 replication in CD4+ T cells in CSF during acute infection in humans

To extend these findings to human HIV-1 infection, which is generally characterized by more robust HIV-1 RNA levels in both plasma and CSF than that observed in this macaque SHIV model, we performed a similar analysis of acute HIV-1 infection in humans. We selected six participants from the RV254 early acute HIV-1 infection cohort in Bangkok, Thailand, with well-characterized viremia. Individuals identified in Fiebig stages III and IV, which correspond to peak HIV-1 RNA levels in plasma and CSF [6], respectively, were prioritized for optimal detection of cell-associated virus (**Table 1**). These Fiebig stages represent an estimated 14 and 19 days post-infection, respectively [24]. Five additional participants were included in additional analyses described below. As previously reported in this cohort, CSF HIV-1 RNA ranged from 10^3^−10^5^ copies per mL (N = 11), which was ~2–3 logs lower than that in plasma.

**Fig 4:**
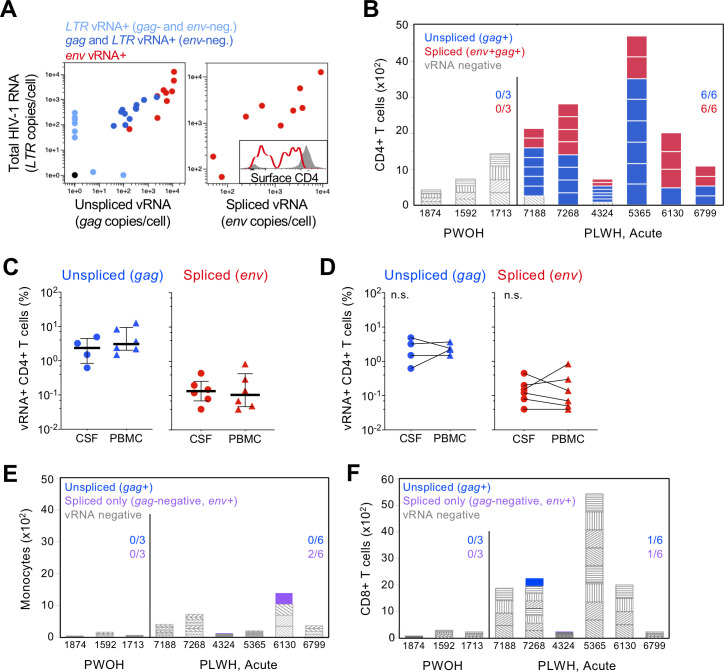
HIV-1 infection of CD4+ T cells in CSF during acute infection in humans. (A) Single-cell analysis of vRNA content in memory CD4+ T cells from PBMC of an acutely infected PLWH from the RV254 cohort. CRF01_AE HIV-1 mRNA was measured by RT-qPCR using assays specific for LTR, *gag*, and *env* in cells FACS sorted at one per well. Each symbol depicts vRNA content for one cell. Symbol color represents the combination of vRNAs detected in the cell, as indicated in the key. Surface CD4 protein expression for *env* vRNA+ cells (red line) was measured by flow cytometry and is indicated relative to vRNA-negative cells (gray) in inset. (B) HIV-1 vRNA RT-qPCR positivity among CD4+ T cells isolated by flow cytometry from CSF of PWOH and people living with acute HIV-1. Bars indicate the number of cells analyzed for each individual; stacked bars reflect the size of each replicate pool of CD4+ T cells. Bar coloring indicates HIV-1 vRNA positivity for unspliced (blue, *gag*) or spliced plus unspliced (red, *env* and *gag*) mRNA; vRNA-negative replicates are colored gray. (C) Distribution of HIV-1 *gag* (left) and *env* (right) vRNA+ CD4+ T cell frequency in CSF and PBMC is shown for individuals represented in (B). HIV-1 *gag* vRNA+ CD4+ T cell analysis in CSF was limited to four individuals. Bars indicate median values and whiskers span the interquartile range. (D) Paired analysis comparing the infected CD4+ T cell frequency between CSF and PBMC is shown. Significant differences between tissues was assessed using Wilcoxon matched-pairs rank test. (E) HIV-1 vRNA positivity among monocytes and CD8+ T cells (F) isolated from CSF as in (B); purple, *env+* and *gag*-negative. Fraction of individuals with vRNA+ cells is indicated.

To measure HIV-1-infected cells in the CNS, CSF CD4+ and CD8+ T cells and monocytes were FACS sorted as described above followed by RT-qPCR for HIV-1 *gag* and *env*, using assays optimized for CRF01_AE detection. Assay validation for sensitivity and specificity to detect active viral replication was performed on CD4+ T cells infected *in vivo* from participants of the RV254 cohort. CD4+ T cells positive for spliced *env* vRNA contained 10^3^−10^4^ copies of unspliced (*gag*) HIV-1 RNA, ~10-100-fold more than vRNA+ cells lacking spliced transcripts, and downregulated surface CD4 protein, providing direct evidence of abundant viral transcription and viral protein expression in cells identified by our spliced vRNA assay (**Fig 4A**). In addition, control experiments confirmed that the majority of vRNA detected was not derived from virions bound to the cell surface (**S2B and S2C Fig**). Strikingly, transcriptionally active CD4+ T cell infection (*env+gag+)* was observed in all acute infection CSF specimens surveyed (**Fig 4B**). The frequency of productively infected cells ranged from 0.04%-0.45% of CSF CD4+ T cells (**Fig 4C and 4D**). *gag* vRNA+ cells were even more abundant and finer resolution cell sorting was required to determine their frequency (**S4A Fig)**, estimated at 0.6%-4.9% of CD4+ T cells. Thus, on average >1% of CD4+ T cells in the CSF harbored vRNA during acute infection and approximately 10% of these cells supported virus replication as evidenced by spliced vRNA expression. CSF cells from PWOH were negative for vRNA. Cell-free CSF supernatant HIV-1 RNA levels did not correlate with the frequency of infected CSF CD4 T cells (*gag+* or *env+gag+*) in this subset of six individuals. We found minimal evidence of infection in CSF monocytes, while CD8+ T cells also rarely contained vRNA (**Fig 4E and 4F**). As in the macaque model, monocyte sampling depth was limited due to their rarity in CSF [36].

A similar analysis of memory CD4+ T cells in PBMC specimens from the same individuals was performed to compare the infected cell frequency between CSF and PB. HIV-1 replication active (*env+gag+*) infected CD4+ T cells comprised 0.04–0.84% of the memory compartment in PBMC (**Fig 4C**), while inactive infection (*env*-negative, *gag*+) again averaged approximately ten-fold higher, ranging from 1.5%-12.6%. These values did not significantly differ from those observed in the matched CSF specimen (**Fig 4D**). CD4+ T cell infection in PB was not strongly correlated with that in CSF, for either *gag+* only or *env+gag+* cells, in this small sample set. Overall, both non-productive and productive CD4+ T cell infection was robust in both CSF and PB during acute HIV-1 infection.

### Lymphocyte and monocyte activation in CSF and PB during acute HIV-1 infection

Prior study in this cohort identified dynamic alterations in CD8+ T cell responses in the CSF evolving over Fiebig stages in acute HIV-1 infection [35]. To examine the broader cellular composition of CSF in AHI, including CD4+ T cells and monocytes, we explored the relative proportion of different cell types in the CSF by flow cytometry. The frequency of monocytes in CSF was similar between PWOH and people living with acute HIV-1 infection (N = 5 and 9, respectively; **S4B Fig**). CD8+ T cell frequency increased during infection, as previously described [35], while CD4+ T cells trended lower, resulting in diminished CSF CD4+/CD8+ T cell ratios (**S4B and S4C Fig**). PBMC CD4+/CD8+ T cell ratios were also reduced. CD4+ T cells in both CSF and PBMC displayed a predominantly memory phenotype, based on surface protein marker expression, and memory cell frequency did not differ between acute infection and PWOH (**S4D Fig**).

**Fig 5:**
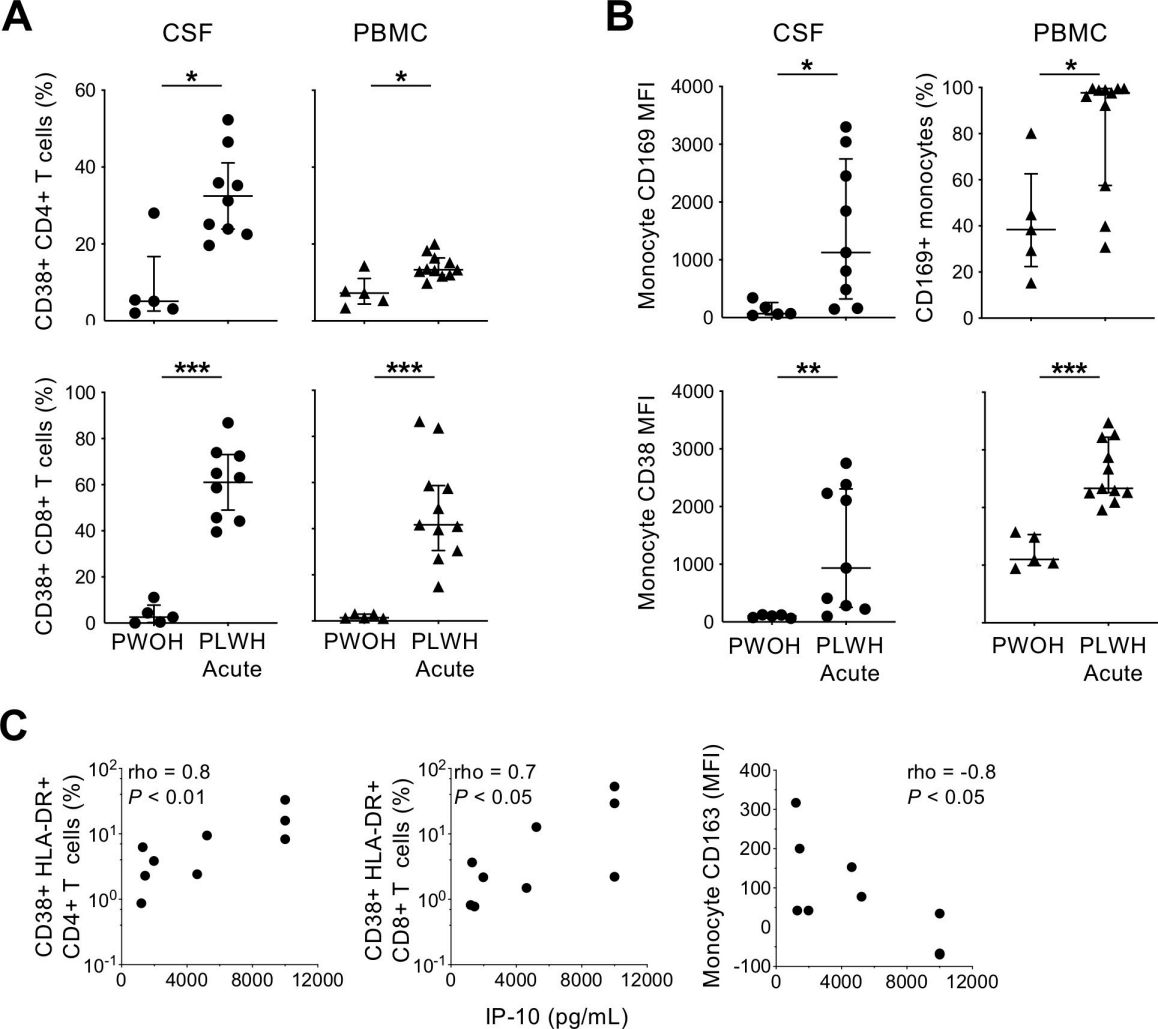
Cellular activation in CSF during acute HIV infection. (A) CD38+ T cell frequency was measured by flow cytometric detection of surface staining on CD4+ (top) and CD8+ (bottom) T cells in CSF (left) and PB (right) of PWOH and PLWH in acute infection. (B) Monocyte activation measured by CD169 (top) and CD38 (bottom) surface staining and flow cytometry in CSF (left) and PB (right). Significant differences by Mann-Whitney test are indicated as follows: *, P <0.05; ** P <0.01; *** P <0.0001. (C) Correlation between CSF IP-10 concentration and activated CD38+HLA-DR+ CD4+ T cells (left), CD38+HLA-DR+ CD8+ T cells (middle), and CD163 surface expression by monocytes (right) in CSF of PLWH. Rho and P values indicate Spearman correlation.

Since acute HIV-1 infection stimulates systemic immune activation and antiviral activity, we sought to determine whether monocytes and T cells in CSF also exhibit heightened activation. The frequency of activated CD4+ T cells, as measured by surface CD38 expression, increased during acute infection in both CSF and PBMC compared to PWOH (*P<*0.05; **Fig 5A**). Median activated CD4+ T cells increased by ~6-fold in CSF and ~2-fold in circulation, indicating considerable HIV-1-associated T cell activation within the CNS that may exceed that in PB. CD8+ T cells were also highly activated in CSF, consistent with prior analysis [35], as well as in PB. Similar results were observed for T cells co-expressing HLA-DR and CD38 (**S5A and S5B Fig**). CSF monocytes also exhibited marked activation during acute HIV-1 infection, with elevated surface expression of both CD169 and CD38 (**Fig 5B**). Median fluorescence intensities increased ~16- and 9-fold for CD169 and CD38, respectively. Monocyte activation marker expression was also greater in PB, consistent with prior studies. Taken together, both CD4+ and CD8+ T cells as well as monocytes were highly activated in CSF during early acute HIV-1 infection.

As has been previously reported in this cohort [6,7,37], multiple soluble markers of inflammation were differentially expressed in CSF during acute HIV-1 infection. Among a panel of 44 pro-inflammatory cytokines and biomarkers measured by Luminex and ELISA in this subset of nine individuals, IL-10 (*P<*0.01), IP-10 (*P<*0.01), neopterin (*P<*0.01) and sCD163 (*P<*0.05) were ~2-5-fold elevated in CSF compared to PWOH. Exploring the relationship between CSF cellular activation and these pro-inflammatory factors, CD4+ and CD8+ T cells co-expressing CD38 and HLA-DR were positively correlated with IP-10 (rho = 0.8, *P<*0.01; and rho = 0.7, *P<*0.05 respectively), while monocyte CD163 expression (cleaved from cell surface during activation) was negatively correlated (rho = -0.8, *P<*0.05) (**Fig 5C**). No associations were observed for the other biomarkers. Infected CD4+ T cell frequency in CSF did not correlate with the elevated factors among the six individuals for whom frequency estimates were made, though replication-active CD4+ T cells were associated with vascular endothelial growth factor C levels (rho = 0.8, *P*<0.05), an angiogenesis factor with known deleterious effects in the CNS [38], a finding that bears investigation in a larger study. These data indicate that activated leukocytes are associated with a pro-inflammatory response in CSF during acute HIV-1 infection.

## Discussion

Early HIV-1 entry into the CNS has the potential to establish distinct viral reservoirs of infected cells as well as initiate inflammatory processes that may contribute to long-term cognitive impairment in PLWH on suppressive ART. Our studies provide direct evidence of CD4+ T cell infection in CSF during early acute infection in two independent models–macaque R5-tropic non-accelerated SHIV infection and human HIV-1 infection. CSF CD4+ T cell infection was consistently detected in PLWH and at frequencies ranging from ~0.03–3% of T cells. Infected cell burden was similar between CSF and other anatomical sites, i.e. PB and lymph nodes, suggesting infected cell trafficking and possible equilibration between these compartments. Both T lymphocytes and monocytes were highly activated in CSF during acute HIV-1 infection, indicating broad immune stimulation. Taken together, these findings suggest active viral replication occurs in T cells within the CNS in acute infection concurrent with a robust inflammatory response.

Some models of HIV-1 early entry into the CNS suggest that systemic inflammation disrupts the blood-brain barrier, allowing plasma virions or blood cells harboring virus to traffic into the CNS [39,40]. Circulating monocytes have been implicated in this process due to detection of infected perivascular macrophage/microglia in the CNS of macaques infected with highly pathogenic SIV. However, increased migration of activated T cells across an intact blood-brain barrier is also possible and is supported by low-level T cell infiltrate reported in brain parenchyma of macaques infected with less aggressive SHIV and with intact barriers [16]. Here, we measured cellular infection in CSF during early acute infection directly *ex vivo* and observed widespread CD4+ T cell infection in all six PLWH and the majority of macaques, including evidence of active viral replication. These findings are consistent with sequence analysis of CSF viral variants during later stages of acute HIV-1 infection in humans, which identified primarily R5 T cell-tropic virus [15]. Moreover, they suggest that early HIV-1 RNA detected in the CSF is likely derived from infected CD4+ T cells residing in the CNS, rather than free virus transiting from the blood. A correlation between CSF T cell infection frequency and CSF HIV-1 RNA in future well-powered studies would support this interpretation.

CD4+ T cells hosting active viral replication in the CNS during acute infection may provide an early opportunity for establishing reservoirs in non-lymphoid or neuronal cells. For example, cell-to-cell contact between infected CD4+ T cells and astrocytes, the most abundant and long-lived cell type in the brain, results in efficient HIV-1 transmission [41]. While we did not see evidence of monocyte infection in CSF (or PB) during acute infection, we were unable to probe this population in depth due to limited numbers of monocytes in CSF from both macaques and humans. Given the well-established role of myeloid lineage cells in supporting HIV-1 infection and replication in the brain during chronic infection [42–47], extending this analysis to chronic infection when myeloid cells may be more likely to harbor virus will aid in understanding their contribution to CNS pathogenesis and reservoirs.

Similar infected T cell burden between CSF and PB (and lymph nodes in macaques) during acute infection suggests equilibrated cell-associated viral replication across these anatomical sites. Genetic evidence of independent HIV-1 replication in the CNS relative to plasma as assessed by viral genomic sequencing has also been limited in primary infection [13–15], in contrast to later disease stages. In analyses of primary infection conducted to date, most individuals exhibit equilibrated HIV-1 sequences between blood and CSF compartments, though cases of compartmentalized CSF virus have been observed in the settings of multiple transmitted/founder viruses and beyond four months of infection. Thus, the compartmentalized HIV-1 variants detected in brains and CSF of individuals with HIV-1-associated dementia most likely reflect a process initiated after initial establishment of viral infection. Comparable CD4+ T cell infection between PB and CSF is consistent with a ~2–3 log_10_ difference in cell-free HIV-1 RNA levels between these compartments, as the CD4+ T cell concentration in CSF is ~10^2^−10^4^-fold lower than that of PB [36]. Similar activation profiles of PB and CSF leukocytes also suggest shared inflammatory states between these sites. Taken together, our data are consistent with cell trafficking from the blood into the CSF, resulting in equilibration of infected and activated cells between these compartments during early acute infection. Related analyses of HIV-1 reservoirs in treated and untreated chronic PLWH reported a positive correlation between HIV-1 DNA levels in CSF cell pellets and HIV-1 DNA in PBMC [48], indicating that similar infected cell burdens across compartments in early infection reported here may extend to later stages as well.

Leukocyte activation and viral replication within CSF during acute HIV-1 infection likely contribute to CNS inflammation, immunopathology and HIV-1 replication [49]. In addition to increased CD8+ T cell activation as described previously [35,50,51], we report heightened activation of CSF CD4+ T cells and monocytes during acute HIV-1 infection. CSF CD4+ and CD8+ T cells were also activated during acute SHIV infection. Activated cells are more permissive to HIV-1 infection and secrete soluble inflammatory markers that increase in the CSF during early infection and are associated with neuronal injury [7,16,37]. Direct evidence of this was observed in CSF during acute HIV-1 infection in our study through elevated pro-inflammatory chemoattractants IL-10, IP-10, neopterin, and sCD163, consistent with prior reports [6,7,37], and correlation between IP-10 concentration and activated CD4+ and CD8+ T cells and monocytes. Taken together, these findings implicate these leukocytes in CNS inflammation and pleocytosis [38,52–54]. We did not observe an association between cellular activation and T cell infection levels during acute SHIV infection, as has been reported previously in chronic HIV-1 infection by some studies [55–57]. One potential implication of this discrepancy is that cellular activation seen later in infection may be a consequence of the infected cell burden, with the important caveat that these studies do not establish causality. It is also possible CD38 expression alone may not be optimal for detecting this association, as prior associations were found primarily with HLA-DR+ CD4+ T cells and not CD38 [58].

The transient nature of PB monocyte activation and absence/low level of CSF monocyte activation in the SHIV macaque model were unexpected observations, as monocyte activation has been observed during both acute and chronic SIV infection [20,59–61]. However, blood monocyte activation appears to be closely linked to uncontrolled viremia [60], and biphasic activation was reported in some SIV models, with peak activation occurring 1–2 weeks PI followed by resolution for several weeks and then another increase prior to AIDS onset [20,62]. This pattern is not inconsistent with our findings. Evaluation of additional monocyte activation markers in CSF is warranted to corroborate our CD169 findings. It is also possible that the more pathogenic nature of most SIV strains relative to SHIV-1157ipd3N4 may heighten innate immune activation.

The rhesus macaque SHIV model employed here, in two independent facilities, recapitulated several aspects of acute HIV-1 infection in humans, supporting its utility for studying early HIV-1-associated neuropathological events [16]. Widespread CSF cellular infection and immune activation, for example, were observed in both models. However, these findings were overall more prominent in people, likely due to sampling at approximately peak viral replication in plasma and CSF, while macaque CSF analyses were performed several weeks following peak plasma viremia and in the context of limited CSF viremia. Therefore, the SHIV model data reported here may underestimate events occurring earlier in the course of infection. Larger CSF cell collections from humans also enabled greater sampling depth for detection of infected cells. Few other SIV/SHIV strains have been shown to resemble early CSF findings of acute HIV-1 infection in humans. Neurovirulent SIV clones are the best characterized in the CNS compartment and while they achieve more robust viral RNA in CSF of macaques than the SHIV strain studied here [19,21], these strains exhibit accelerated neurologic disease similar to end stage disease and do not faithfully recapitulate the more subtle neurologic changes of early/acute HIV-1. Development of CNS infection models using SHIVs expressing transmitted-founder HIV-1 envelopes may more closely mimic acute HIV-1 infection in humans.

These findings add to a growing body of evidence that CD4+ T cell infection plays a central role in early viral entry of the CNS. An important implication of these findings is that even very early ART initiation may not be able to prevent establishment of CNS viral reservoirs and sources of long-term inflammation. Future studies evaluating the impact of ART on infected cell burdens in CSF will address the extent to which cell-associated virus persists during suppressed infection and investigate associations with persistent neurologic and cognitive abnormalities observed in humans despite suppressive ART.

## Supporting information

S1 Fig Flow cytometry gating strategy for sorting cells from CSF, PB and lymph nodes. **(A)** Flow cytometry cell sorting gating tree used to isolate CD4+ T cells, CD8+ T cells, and monocytes from CSF. **(B)** Flow cytometry cell sorting gating tree used to isolate monocytes and memory CD4+ T cells from PBMC and LNMC specimens. Serial gating was applied from left to right for monocytes (top row) and CD4+ T cells. Memory CD4+ T cell gating markers used for rhesus (middle) and human (bottom) are shown.
(TIFF)

S2 Fig Validation of viral RNA RT-qPCR assays for SHIV-1157ipd3N4 and CRF01_AE HIV-1. **(A)** Linear performance and sensitivity of SHIV RNA RT-qPCR assays. SHIV *env/vpu/nef* and SIV *gag* assays were validated by RT-qPCR on serially diluted RNA isolated from rhesus macaque PBMC infected with SHIV-1157ipd3N4 *in vitro*. PBMC were stimulated with PHA for four days followed by in vitro infection culture for three days. Graphs depict Et (40-Ct) values obtained for eight replicates of nine serial two-fold RNA dilutions corresponding to 3–1000 cells by mass. **(B)** The frequency of *gag* vRNA+ memory CD4 T cells in PBMC following incubation in the presence or absence of trypsin. PBMC from two RV254 participants in acute untreated HIV-1 infection were treated with trypsin, followed by memory CD4 T cell isolation by gating on CD8-negative CD3+ lymphocytes and CD45RO+CD45RA-negative cells, limiting dilution cell sorting, and viral RNA RT-qPCR to estimate infected cell frequency. **(C)** Representative cell surface CD4 expression measured by flow cytometry for PBMC described in (B).
(TIFF)

S3 Fig Cell-associated SHIV vRNA among CSF CD8+ T cells during acute SHIV infection in macaques. Viral RNA RT-qPCR positivity for FACS sorted CD8+ T cells from CSF of uninfected and SHIV-infected macaques 4, 8, and 12-weeks PI. The number of cells analyzed for each animal is indicated on the y-axis, with stacked bars reflecting the size of each sort replicate. Bar coloring indicates positivity for unspliced only (blue) or spliced (red) SHIV RNA; viral RNA negative replicates are colored gray.
(TIFF)

S4 Fig CSF T cell-associated HIV-1 RNA and CSF mononuclear cell composition during acute infection in humans. Viral RNA RT-qPCR positivity for FACS sorted CD4+ **(A)** from CSF of PLW acute HIV-1 and PWOH. The number of cells analyzed for each individual is indicated on the y-axis, with stacked bars reflecting the size of each sort replicate. Replicates consisted of limited cell numbers. Bar coloring indicates positivity for unspliced only (blue) or spliced (red) SHIV RNA; viral RNA negative replicates are colored gray. **(B)** The frequency of T cell subsets and monocytes in CSF determined by flow cytometry is shown for PWOH (N = 5) and PLW acute HIV-1 (N = 9). T cell subset frequency was calculated as the percentage of all T cells; monocytes as the percentage of all viable CSF cells. **(C)** CD4+/CD8+ T cell ratio and the proportion of CD4+ T cells that are memory **(D)** are shown for CSF (left) and PBMC (right). Memory CD4+ T cells were defined as CD45RO+ or negative for both CD45RO and CCR7. Significant differences by Mann-Whitney test are indicated as follows: *, *P* <0.05; **, *P* < 0.01; ***, *P* <0.0001.
(TIFF)

S5 Fig Cellular activation in CSF during acute HIV infection. Frequency of CD4+ **(A)** and CD8+ **(B)** T cells double-positive for CD38 and HLA-DR is shown for CSF (left) and PBMC (right) of PWOH and PLW acute HIV-1. Significant differences by Mann-Whitney test are indicated: *, *P* <0.05; **, *P* < 0.01; ***, *P* <0.0001.
(TIFF)
