# Supplementary figures and images for: Cerebrospinal fluid CD4+ T cell infection in humans and macaques during acute HIV-1 and SHIV infection
Source: PLoS Pathog. 2021 Dec 7;17(12):e1010105. doi: 10.1371/journal.ppat.1010105 (PMC8683024; doi:10.1371/journal.ppat.1010105)

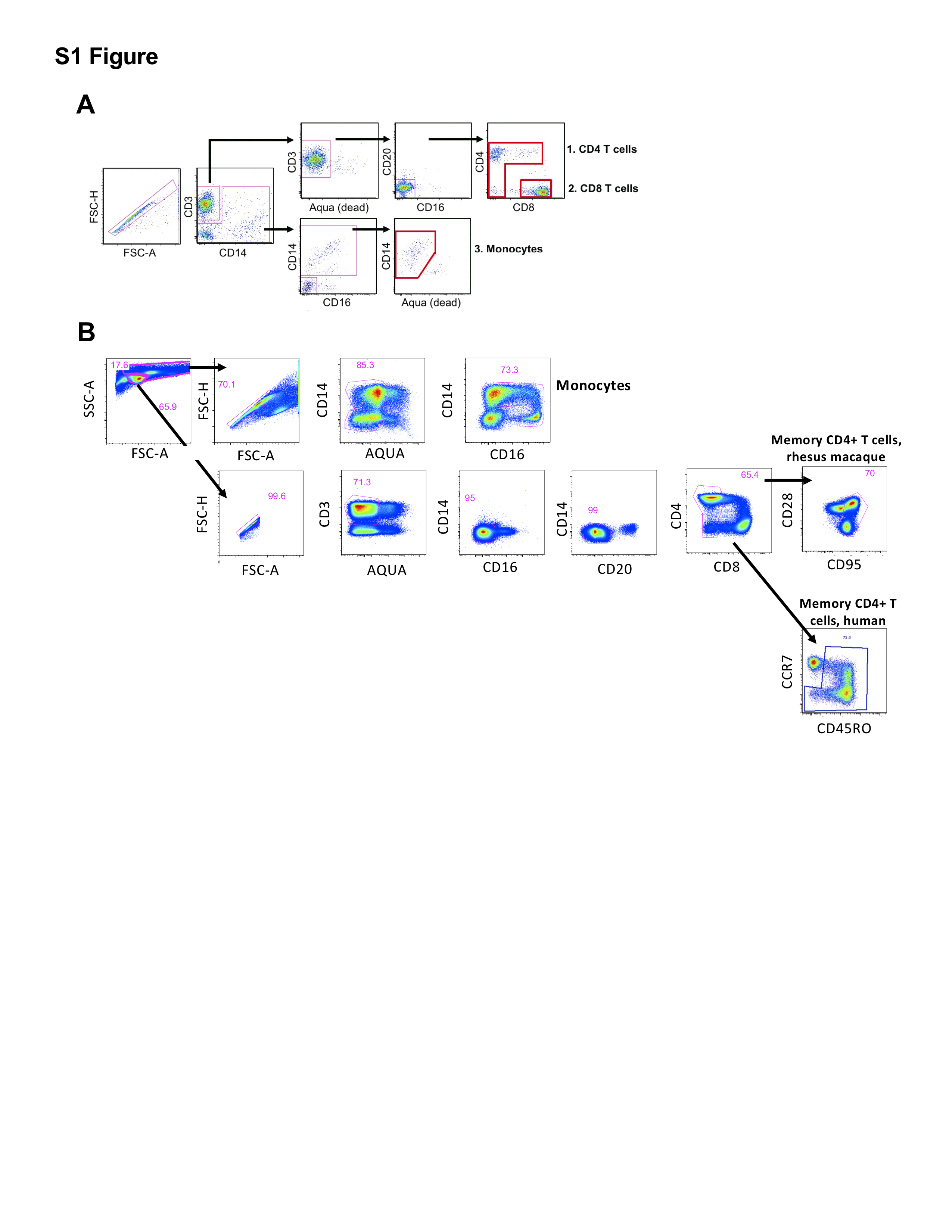

Supplement: S1 Fig — (A) Flow cytometry cell sorting gating tree used to isolate CD4+ T cells, CD8+ T cells, and monocytes from CSF. (B) Flow cytometry cell sorting gating tree used to isolate monocytes and memory CD4+ T cells from PBMC and LNMC specimens. Serial gating was applied from left to right for monocytes (top row) and CD4+ T cells. Memory CD4+ T cell gating markers used for rhesus (middle) and human (bottom) are shown. (TIFF) [file ppat.1010105.s001.tiff]

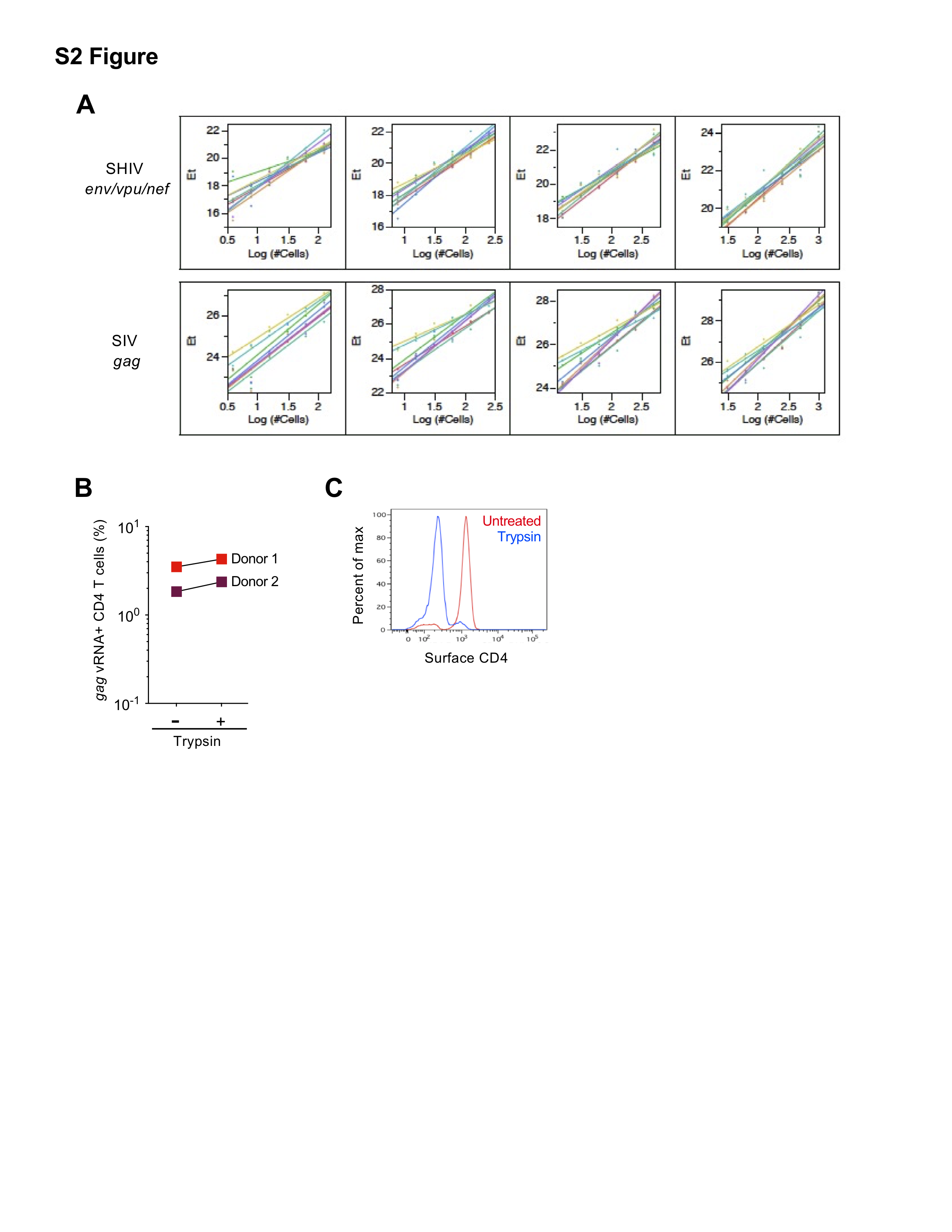

Supplement: S2 Fig — (A) Linear performance and sensitivity of SHIV RNA RT-qPCR assays. SHIV env/vpu/nef and SIV gag assays were validated by RT-qPCR on serially diluted RNA isolated from rhesus macaque PBMC infected with SHIV-1157ipd3N4 in vitro. PBMC were stimulated with PHA for four days followed by in vitro infection culture for three days. Graphs depict Et (40-Ct) values obtained for eight replicates of nine serial two-fold RNA dilutions corresponding to 3–1000 cells by mass. (B) The frequency of gag vRNA+ memory CD4 T cells in PBMC following incubation in the presence or absence of trypsin. PBMC from two RV254 participants in acute untreated HIV-1 infection were treated with trypsin, followed by memory CD4 T cell isolation by gating on CD8-negative CD3+ lymphocytes and CD45RO+CD45RA-negative cells, limiting dilution cell sorting, and viral RNA RT-qPCR to estimate infected cell frequency. (C) Representative cell surface CD4 expression measured by flow cytometry for PBMC described in (B). (TIFF) [file ppat.1010105.s002.tiff]

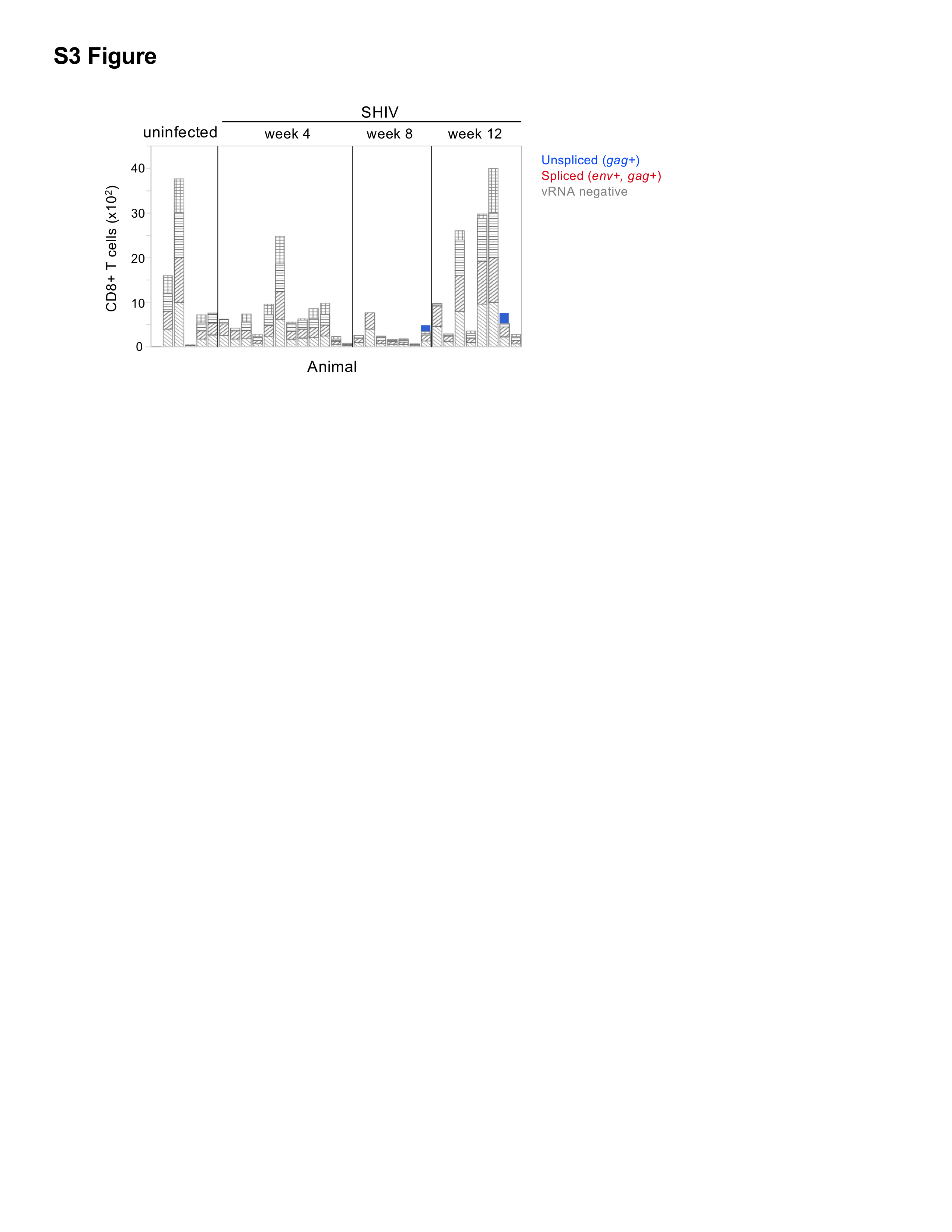

Supplement: S3 Fig — Viral RNA RT-qPCR positivity for FACS sorted CD8+ T cells from CSF of uninfected and SHIV-infected macaques 4, 8, and 12-weeks PI. The number of cells analyzed for each animal is indicated on the y-axis, with stacked bars reflecting the size of each sort replicate. Bar coloring indicates positivity for unspliced only (blue) or spliced (red) SHIV RNA; viral RNA negative replicates are colored gray. (TIFF) [file ppat.1010105.s003.tiff]

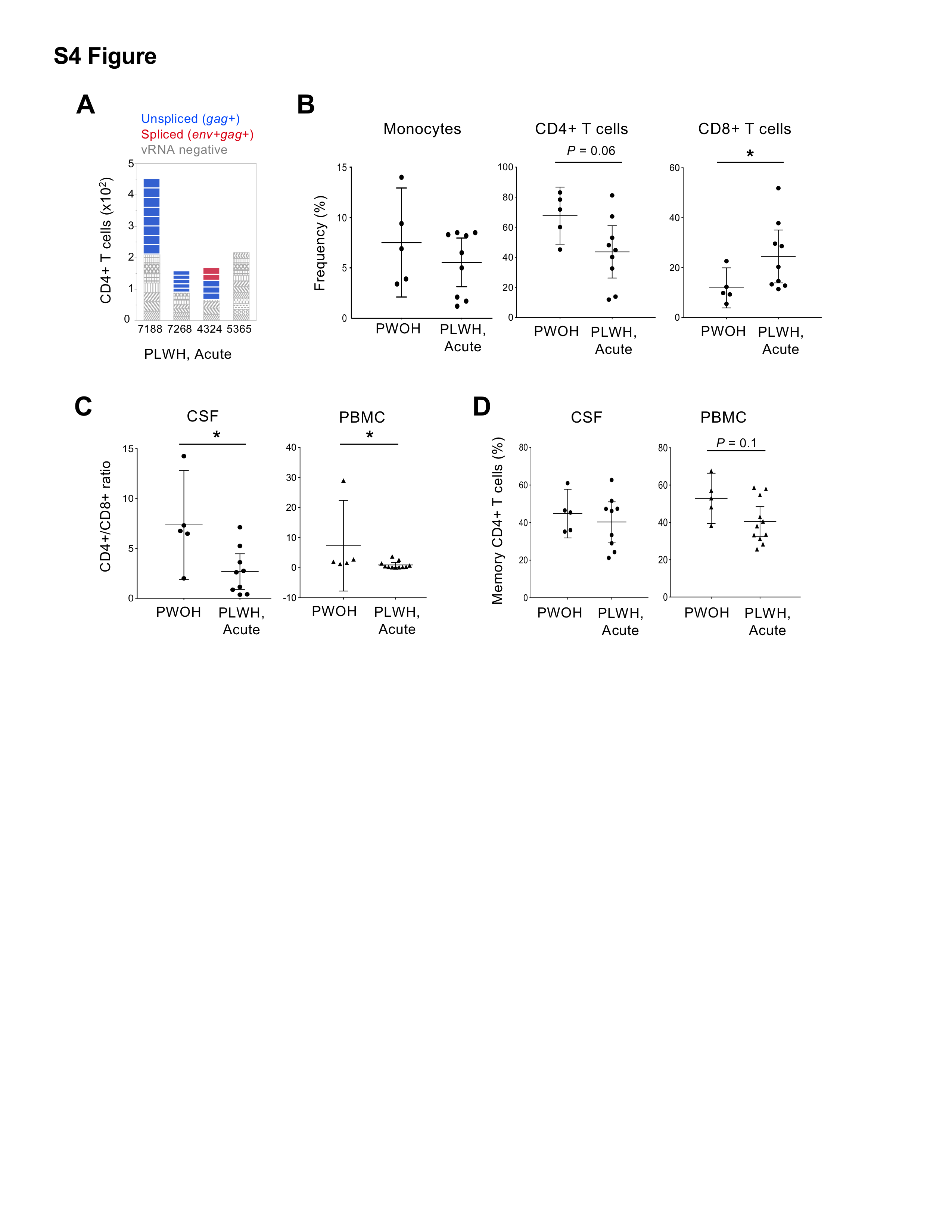

Supplement: S4 Fig — Viral RNA RT-qPCR positivity for FACS sorted CD4+ (A) from CSF of PLW acute HIV-1 and PWOH. The number of cells analyzed for each individual is indicated on the y-axis, with stacked bars reflecting the size of each sort replicate. Replicates consisted of limited cell numbers. Bar coloring indicates positivity for unspliced only (blue) or spliced (red) SHIV RNA; viral RNA negative replicates are colored gray. (B) The frequency of T cell subsets and monocytes in CSF determined by flow cytometry is shown for PWOH (N = 5) and PLW acute HIV-1 (N = 9). T cell subset frequency was calculated as the percentage of all T cells; monocytes as the percentage of all viable CSF cells. (C) CD4+/CD8+ T cell ratio and the proportion of CD4+ T cells that are memory (D) are shown for CSF (left) and PBMC (right). Memory CD4+ T cells were defined as CD45RO+ or negative for both CD45RO and CCR7. Significant differences by Mann-Whitney test are indicated as follows: *, P <0.05; **, P < 0.01; ***, P <0.0001. (TIFF) [file ppat.1010105.s004.tiff]

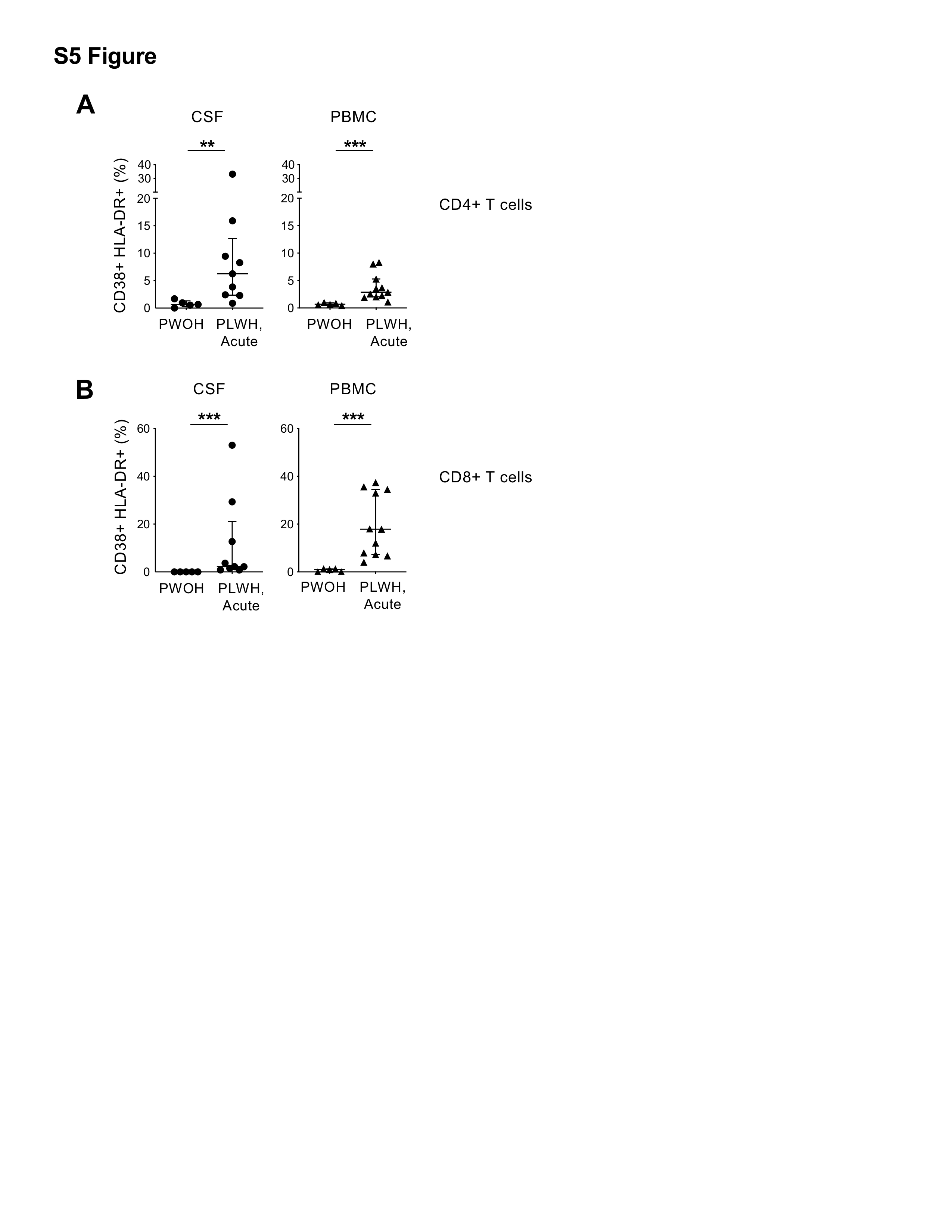

Supplement: S5 Fig — Frequency of CD4+ (A) and CD8+ (B) T cells double-positive for CD38 and HLA-DR is shown for CSF (left) and PBMC (right) of PWOH and PLW acute HIV-1. Significant differences by Mann-Whitney test are indicated: *, P <0.05; **, P < 0.01; ***, P <0.0001. (TIFF) [file ppat.1010105.s005.tiff]
